# Site-specific pharmaco-laser therapy: A novel treatment modality for refractory port wine stains

**Published:** 2019-05-01

**Authors:** M. Ingmar van Raath, Jojanneke E. van Amesfoort, Martin Hermann, Yasin Ince, Maurice J. Zwart, Agustina V. Echague, Yan Chen, Baoyue Ding, Xuan Huang, Gert Storm, Michal Heger

**Affiliations:** ^1^Department of Pharmaceutics, College of Medicine, Jiaxing University, Jiaxing, Zhejiang, PR China; ^2^Department of Experimental Surgery, Amsterdam UMC, University of Amsterdam, Amsterdam, the Netherlands; ^3^Department of Anesthesiology and Critical Care Medicine, Medical University of Innsbruck, Innsbruck, Austria; ^4^Wellman Center for Photomedicine, Massachusetts General Hospital, Boston, Massachusetts, USA; ^5^Department of Clinical Medicine, College of Medicine, Jiaxing University, Jiaxing, Zhejiang, PR China; ^6^Department of Pharmaceutics, Utrecht Institute for Pharmaceutical Sciences, Utrecht University, Utrecht, the Netherlands; ^7^Department of Controlled Drug Delivery, MIRA Institute for Biomedical Technology and Technical Medicine, University of Twente, Enschede, the Netherlands

**Keywords:** antifibrinolytics, thermal coagulum, hemodynamics, primary and secondary hemostasis, mild hyperthermia, liposomes, photosensitizer, procoagulants, steric stabilization, tranexamic acid

## Abstract

Despite extensive efforts to optimize laser therapy, i.e., the current gold standard treatment, a majority of port wine stain (PWS) patients responds suboptimally to laser therapy. This paper describes the niceties of a novel PWS treatment modality termed site-specific pharmaco-laser therapy (SSPLT). In contrast to the classic approach of enhancing the extent of intravascular photocoagulation (the photothermal response), SSPLT focuses on optimization of post-irradiation thrombus formation (i.e., the hemodynamic response) by combining conventional laser therapy with the administration of thermosensitive drug delivery systems that encapsulate prothrombotic and antifibrinolytic drugs. The aim of SSPLT is to instill complete lumenal occlusion in target vessels, which has been linked to optimal PWS blanching.

**Relevance for patients:**

The current treatment options for PWS patients are limited in efficacy. Novel therapeutic modalities are needed to more effectively treat patients with recalcitrant PWSs. SSPLT is an experimental-stage treatment modality that could serve as an adjuvant to pulsed dye laser therapy for a selected group of patients whose PWS is ill-responsive to standard treatment. The expected clinical result of SSPLT is improved lesional blanching.

## 1. Introduction

### 1.1. Port wine stains

**Figure 1:**
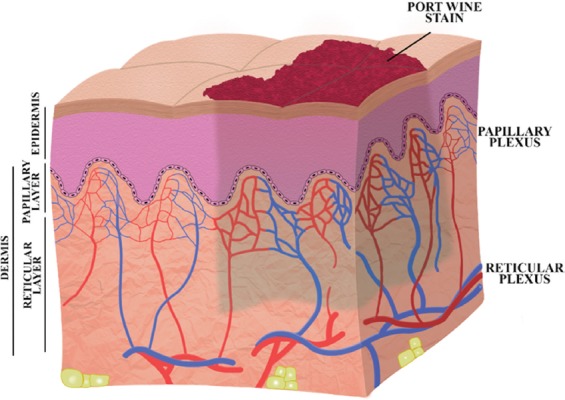
A schematic cross-section of skin with a port wine stain. The characteristic appearance of the skin is caused by hyperdilated capillaries and post-capillary venules mainly in the papillary plexus, which contain a large fraction of blood and hence cause the affected portion of the skin to appear pink to red.

Port wine stains (PWSs) are congenital vascular lesions characterized by hyperdilated capillaries and post-capillary venules (typically 30–300 μm in diameter [1]) in the papillary and mid-reticular layers of the dermis (Figure 1). These birthmarks occur in 0.3–0.5% of infants and initially appear as flat, pink macules that gradually progress into hypertrophic, red-to-purple lesions [2-5]. Although the exact etiological origin remains unknown, studies observed low neural density at the periphery of the ectatic vessels, which may account for inadequate neurotrophism and tonus regulation of the affected vasculature, and overexpression of vascular endothelial growth factor (an inducer of both proliferation and vasodilation) and its receptor [6,7]. Genetic alterations, most importantly somatic mutations in the *GNAQ* gene encoding the guanine nucleotide-binding protein G alpha-q, imply a genetic origin [8-13]. Tan et al. demonstrated the expression of endothelial progenitor cell markers and co-expression of the arterial and venous markers ephrin B2 (EfnB2) and Eph receptor B1 (EphB1), respectively, in PWS vessels [14]. The Efn-Eph family is a group of widely expressed ligands and receptors capable of forward and backward signaling that mediate tissue morphogenesis and cell differentiation, including establishment of arterial-venous vasculature, angiogenesis, and invasion. Corroboratively, co-expression of EfnB2 and EphB1 in the normal human endothelial cells (ECs) led to the formation of PWS-like vessels *in vitro* [14]. Taken together, these findings suggest an impaired endothelial differentiation in PWS vessels. Increased perfusion pressure and age-related collagen degeneration in the dermis are possible contributory factors to the progressive vascular hyperdilation with age [4,15,16].

By the age of 46, two-thirds of the affected individuals have developed papular or nodular components resulting from soft tissue overgrowth, causing dysmorphosis, asymmetry, and occasional spontaneous bleeding [17-19]. Because 70–80% of these birthmarks occur in the head and neck regions, the aberrant cosmetic appearance of PWSs may significantly impede patients’ psychosocial development and well-being and constitutes a considerable factor in the overall treatment of PWSs [20-24]. The anatomical location and dermatomal distribution pattern of trigeminal PWSs (pertaining to the ophthalmic, maxillary, and mandibular branches of the trigeminal nerve located in the respective facial regions) have been linked to an increased probability of ocular and/or central nervous system complications (glaucoma and/or Sturge–Weber and Klippel–Trénaunay syndrome, respectively) [17,25,26].

### 1.2. Standard treatment of port wine stains and clinical outcomes

**Figure 2:**
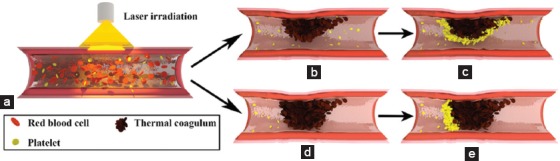
Endovascular laser–tissue interactions in relation to selective photothermolysis are shown in a port wine stain vessel (a) subjected to laser irradiation. During laser irradiation (a), hemoglobin is used as a thermal catalyst to generate intraluminal heat. In this (photothermal) process, supracritical temperatures cause rapid thermal denaturation plasma proteins and blood cell thermolysis, which consequently agglutinate and form a thermal coagulum (b and d). Subsequently, primary and secondary hemostasis are activated and a thrombus develops (hemodynamic response; panel c and e). The photothermal process may result in incomplete (b and c; upper pathway) or complete (d and e; bottom pathway) photocoagulation. Complete photocoagulation of vessels, i.e., the cessation of blood flow by an occlusive thermal coagulum, corresponds to good clinical results (lesional blanching). In contrast, incomplete photocoagulation (b), which can be attributable to several factors such as optical shielding, corresponds to a suboptimal therapeutic effect (no lesional blanching).

The most widely employed therapy for PWSs is non-invasive photocoagulation of the hyperdilated vasculature with a pulsed dye laser (PDL) by selective photothermolysis (SP) (Figure 2) [27]. SP is based on the conversion of radiant energy to heat by hemoglobin (i.e., a mainly blood vessel-confined chromophore), which results in thermal denaturation of blood and, depending on the extent of heat diffusion and convection, the vascular wall and perivascular tissue [1,28-32]. For SP, the pulse duration should be shorter than the thermal relaxation time (i.e., the time required for heated matter to lose 50% of its peak thermal energy through thermal conductivity [33,34]) of the target structure. The hyperdilated blood vessels associated with PWSs have lower surface-to-volume ratios and therefore longer thermal relaxation times and higher thermal masses compared to normal-sized capillaries and post-capillary venules [1,28-32]. Consequently, laser irradiation generates denaturing temperatures in PWS vasculature but not the normal microcirculation.

**Figure 3:**
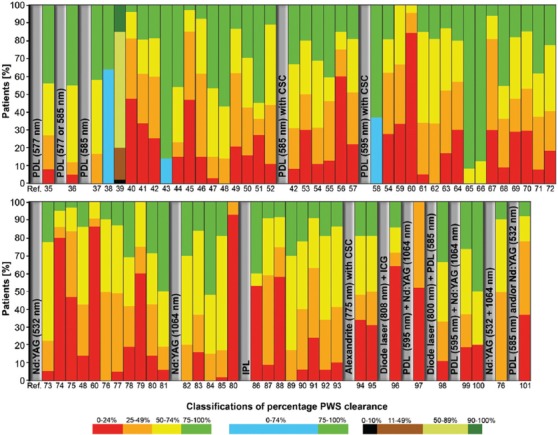
Summary of clinical study outcomes of port wine stain (PWS) laser- or intense pulsed light therapy from 1986 to present. The vertical axis represents the percentage of patients in the color-coded classes with differential levels of percentage PWS clearance indicated in the legend (bottom). Each vertical bar represents an entire study population of one study referenced below the bar. The bars are grouped according to treatment modality. The complete data set is available in Supplemental Table S1. Abbreviations: CSC: cryogen spray cooling, ICG: indocyanine green, Nd:YAG: neodymium:yttrium-aluminum garnet, PDL: pulsed dye laser, PWS: port wine stain, Ref.: reference.

Although the selectivity of SP toward PWS vasculature versus normal vasculature is generally good in the clinical setting, treatment outcomes of PDL therapy are relatively poor (Figure 3, [35-101]; Supplemental Table S1). This can be ascribed to insufficient heat generation in a portion of the vessels and hence incomplete photocoagulation of the target structures [102,103]. Clinically, complete photocoagulation of the vascular lumen (Figure 2, panels d and e) is associated with well-responding lesions [28], which corresponds to approximately 40% of cases. In contrast, moderately responding (20–46%) and refractory PWSs (14–40%) are characterized by dermal vasculature comprised of incompletely photocoagulated blood vessels (Figure 2, panel b and c) [1,28-32,55,104]. Accordingly, the goal of PDL treatment of PWSs is to achieve complete photocoagulation of the hyperdilated blood vessels.

### 1.3. Causes of therapeutic recalcitrance

The efficacy of SP relies on a series of uncontrollable intrinsic factors, such as epidermal pigmentation, optical shielding by blood and superimposed vessels [1,28,29,105-107], and PWS anatomy [1,28,106-109] and morphology, which is age-dependent [1,28,106-110]. Accordingly, extensive melanin content (corresponding to high Fitzpatrick skin phototypes), high vascular density and superimposition, and large-diameter and deeply-situated vessels altogether contribute to reduced treatment efficacy inasmuch as these factors decrease the penetration of laser light in the skin and PWS vessels and, therefore, decrease intraluminal heat production. In case of deeply situated or optically shadowed vessels, photocoagulation may even be forestalled entirely.

Most of the hyperdilated PWS vasculature is located within ~0.6 mm from the basal membrane [1,4] (i.e., ~0.7 mm from the skin surface, taking into account an epidermal thickness of ~100 μm [111]). Nevertheless, PWS vessels have also been found in the reticular plexus (Figure 1) up to a depth of 3.7 mm [112,113]. Mathematical modeling revealed that the depth to which hyperdilated vasculature is responsible for the visual appearance of PWSs is 0.6–0.9 mm [114,115]. Theoretically, complete lesional clearance can only be accomplished when the photocoagulation depth equals or exceeds the depth to which the hyperdilated vessels contribute to the skin discoloration. The therapeutic recalcitrance of the majority of PWSs may therefore also be explained by the discrepancy between the required depth to which photocoagulation should occur and the actual depth of photocoagulation, which is up to ~0.65 mm in the human skin (with a mean ± standard deviation depth of 0.37 ± 0.17 mm using 585-nm wavelength and 0.45-ms pulse duration) [1,28]. In PWS skin containing dense vasculature [105,116,117], high melanin content [1,108], and dermal blood [22,108] the photocoagulation depth is further reduced, particularly when large-diameter blood vessels are present. At the above-mentioned laser parameters and a radiant exposure of 6.5 J/cm2, complete photocoagulation was shown to occur in superficial vessels not exceeding 150 μm in diameter [1]. In sum, PDL treatment efficacy is considerably hampered in PWSs containing dense, large, and deeply situated vasculature [105,118].

### 1.4. Photodynamic therapy

**Figure 4:**
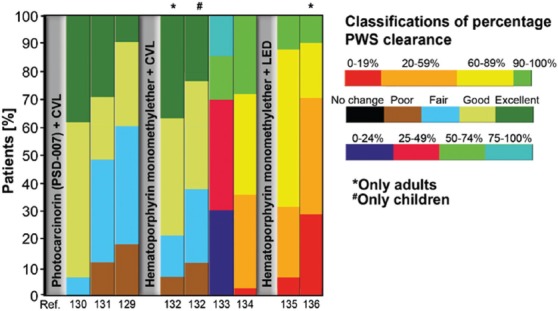
Summary of clinical outcomes of port wine stain (PWS) photodynamic therapy studies from 1990 to present. The vertical axis represents the percentage of patients in the color-coded classes with differential levels of percentage PWS clearance indicated in the legend (right). Each vertical bar represents the entire patient cohort of one study, which is referenced below the bar. The bars are grouped according to treatment modality. The complete data set is available in Supplemental Table S2. Abbreviations: CVL: copper vapor laser, LED: light-emitting diode, PWS: port wine stain, Ref.: reference.

Photodynamic therapy (PDT), which is based on the interaction between an administered photosensitizer and light, has been investigated as an alternative for PDL. Although PDT is primarily known for its application in oncology [119], the treatment is also increasingly being employed for PWS [52,120-123]. After intravenous administration of the photosensitizer, PWSs are irradiated with a laser, similar to PDL therapy. Photosensitizers are molecules that can be brought to an excited and subsequently triplet state by the absorption of resonant light. During electron decay from the triplet state to the ground state, the photosensitizers transfer a portion of the absorbed energy to neighboring molecules, typically molecular oxygen (type II photochemical reaction), to yield singlet oxygen. Alternatively, the triplet state electron is transferred to molecular oxygen or another electron acceptor to yield superoxide anion or a molecular radical, respectively (type I photochemical reaction). All reactive oxygen species (ROS) thus formed are cytotoxic [119,124-126] and thrombogenic [127,128]. This causes EC wall damage, thrombosis, and shutdown of vasculature [129]. Figure 4 summarizes the clinical outcomes of PWS studies using PDT [129-136] (Supplemental Table S2). Most clinical experience with this modality is in China [131,137]. A recent retrospective study found PDT to be as effective as 585-nm PDL (with a 0.30–0.45-ms pulse duration) for pink flat lesions in children and more effective for purple flat lesions in adults [132]. Another study performed in pediatric patients found PDT to be equally effective as 585-nm PDL in red lesions and more effective in purple lesions [52].

Nevertheless, PDT has also failed to achieve good PWS clearance in a substantial portion of patients, further enforcing the medical need to optimize PWS treatment by alternative routes. Robust comparative studies on PDL versus PDT regimens for PWSs are lacking. The main downside of the use of photosensitizers is general photosensitivity, which occurs for days to weeks, depending on the half-life of the photosensitizer used. Long-term adverse effects of PDT for PWSs are, however, rare [138]. Multiple groups are currently attempting to optimize PDT [139] and investigate a combination of PDL with PDT [140-142]. Although such efforts and the implementation of second-generation photosensitizers and treatment protocols are expected to further improve clinical results, complete lesional clearance is unlikely to occur in all PWS patients.

## 2. Endovascular laser-tissue interactions

**Figure 5:**
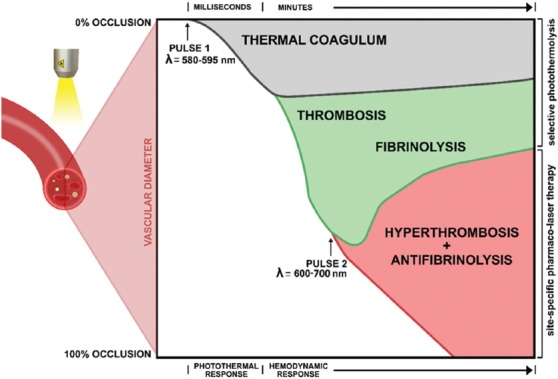
Endovascular laser-tissue interactions in an incompletely photocoagulated blood vessel and the potential of site-specific pharmaco-laser therapy (SSPLT) to modulate part of this process. The extent of occlusion (left y-axis, corresponding to the blood vessel diameter) is plotted against time (top x-axis). The black trace describes the size of a rapidly forming non-occlusive thermal coagulum after initial laser irradiation. The green trace shows normal thrombus formation (the hemodynamic response), which can be divided into a growth phase (thrombosis) and a breakdown phase (fibrinolysis). Both responses are a consequence of selective photothermolysis (right y-axis). The red trace shows the intended effect of SSPLT, namely increased occlusion as a result of locally released procoagulants and antifibrinolytics, which enhance thrombosis and deter fibrinolysis, respectively.

The clinical goal of laser therapy of PWSs, i.e., lightening or preferably complete removal of the lesion, is achieved by the elimination of ectatic PWS vessels and the consequent reduction in abnormally high dermal blood volume (section 1.2.). The endovascular laser-tissue interactions that govern these effects comprise an initial photothermal response [32,143] followed by a hemodynamic response that occurs only in incompletely photocoagulated vessels [144,145], i.e., the blood vessels that are responsible for suboptimal clinical outcomes. The mechanisms underlying the photothermal and hemodynamic response are illustrated in Figures 2 and 5. Figure 5 illustrates how these two fundamental responses can be modulated pharmacologically to enhance vaso-occlusion, and hence clinical outcome, through an experimental modality referred to as site-specific pharmaco-laser therapy (SSPLT). SSPLT is discussed in detail in section 3.

### 2.1. The photothermal response

**Figure 6:**
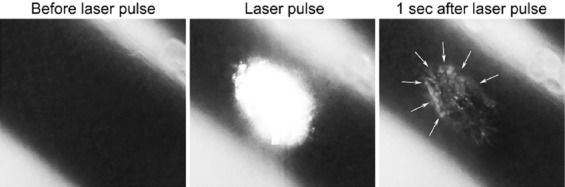
Laser irradiation of a hamster dorsal skin fold venule with a single 30-ms, 532-nm laser pulse results in intraluminal heat generation and the formation of a thermal coagulum (white arrows). During the photothermal response, circulating thermosensitive liposomes (1,2-dipalmitoyl-sn-glycero-3-phosphocholine (DPPC):1,2-distearoyl-sn-glycero-3-phosphoethanolamine-conjugated polyethylene glycol (DSPE-PEG), 10:85:5 molar ratio) loaded with 5(6)-carboxyfluorescein (CF) at a self-quenched concentration (100 mM) were incorporated into the thermal coagulum and released their cargo. Raising the temperature above the phase transition temperature of the main phospholipid component (DSPC, T_m_ = 55.5 °C) results in liposomal membrane permeability and rapid CF release. Part of the CF is trapped within (or tethered to) the thermal coagulum. The CF becomes diluted that in turn causes fluorescence dequenching.

**Figure 7:**
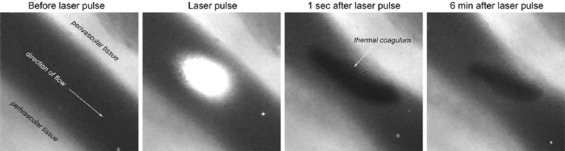
The photothermal response in a hamster dorsal skin fold venule subjected to a single 30-ms, 532-nm laser pulse results in the formation of a thermal coagulum, visible as a black structure (panel labeled “1 sec after laser pulse”). After the end of the laser pulse, thermal coagulum expansion is halted due to ceased heat generation. The thermal coagulum remains tethered to the vascular wall but shrinks in time as a result of shear-mediated deterioration. Experimental data were taken from the study by Bezemer et al. [149].

During laser irradiation, intraluminal heat is generated by laser-targeted hemoglobin (Figure 6) and results in denaturation of plasma proteins (> 45 °C) [146], disruption or even complete disintegration of cell membranes (> 51 °C) of blood cells, specifically red blood cells (RBCs) [147], and thermal necrosis of the vascular wall (> 70 °C). Inasmuch as thermally denatured proteins and disintegrated erythrocytes precipitate, the agglutinated/thermolysed blood subsequently forms an occlusive ’thermal coagulum’ in the lumen at the site of irradiation [1,28,146,148,149]. Furthermore, diffusion of heat into perivascular tissue contributes to vasoconstriction owing to thermal denaturation of collagen [149], the main constituent of the extracellular/perivascular matrix. As the extent of intraluminal heat generation varies (section 1.3), so does the extent of thermal coagulum formation. Moreover, the expansion of thermal coagula is halted in the absence of further energy input, rendering the photothermal response temporally static from the end of the laser pulse onward. Thermal coagula cannot expand after the laser pulse and can only gradually decrease in size by shear-mediated deterioration or disappear completely upon coagulum dislodgment (Figure 7) [149,150]. In case of complete photocoagulation, i.e., when thermal coagula span the entire inner vascular diameter, prolonged cessation of blood flow and widespread thermal damage of the vascular wall cause ischemia and necrosis. Ultimately, these biological reactions to laser irradiation drive the removal of PWS blood vessels and corollary blanching of the lesion [144,103]. However, in refractory PWSs (i.e., those responding suboptimally to laser therapy), photocoagulation is incomplete, which triggers the hemodynamic response [150].

### 2.2. The hemodynamic response

The hemodynamic response, illustrated in Figures 2 and 5, is characterized by the activation of both primary and secondary hemostasis. Thromboembolic activity is located on the thermal coagulum surface, as shown previously [150]. Denaturated proteins that partly make up the coagulum likely contribute to hemostasis by activation of platelets through the constitutively expressed platelet receptor CD42b, even under low shear conditions, and the contact activation pathway (FXII) [149,150]. Several other prothrombotic factors have been described [144,150]. Thermolysis of RBC membranes leads to the release of endogenous adenosine diphosphate (ADP) and exposure of anionic phosphatidylserine (PS), which triggers platelet activation, aggregation [151], and the coagulation cascade [152,153].

Moreover, both primary and secondary hemostasis are activated at sites of coagulum dislodgment. Thermal damage to, or denudation of the endothelial monolayer and the subsequent exposure or expression of tissue factor (TF) and subendothelial matrix constituents presumably are the main responsible triggers. The immobilized endothelial surface forms an ideal anchor for the formation of a durable hemostatic plug. Intact, yet “photostimulated” endothelial membranes also induce platelet tethering, however in a transient manner that eventually results in thrombus dislodgment [154]. The fact that no cases of clinical complications caused by thrombosis or embolization after laser therapy have been reported is probably due to the small size of these thrombi. Neutrophils may potentiate thrombosis by releasing nuclear material known as neutrophil extracellular traps, resulting in FXII activation and inactivation of TF pathway inhibitor (TFPI) (the main TF inhibitor) [155] or by binding of TF-expressing neutrophils to the injured endothelial wall [156]. Moreover, monocyte (MC)-derived microparticles may facilitate thrombosis by delivering additional TF [157].

**Figure 8:**
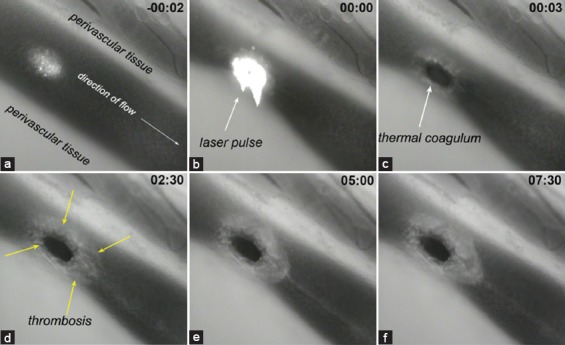
The dynamic nature of laser-induced thrombosis is shown in a hamster dorsal skin fold venule that had been subjected to a single 30-ms, 532-nm laser pulse. Directly after the laser pulse (b) a thermal coagulum is formed (c, black structure). Thermal coagulum formation triggers thrombosis (d, yellow arrows). The thrombus continues to grow (panel c-h) but is also subject to shear-mediated degradation and fibrinolysis. The time relative to the laser pulse is shown in the upper right corner (min:sec). Experimental data were taken from the study by Bezemer et al. [149].

The process of laser-induced thrombus formation is dynamic as opposed to the photothermal response (Figure 8). Previous studies showed that thrombi undergo an initial, predominantly thrombotic growth phase followed by a predominantly fibrinolytic (tertiary hemostasis) shrinking phase. In a hamster dorsal skin-fold model [145], rapid thrombus growth was observed during the first 1.25 min, with thrombus growth continuing, however, at a slower rate, in the subsequent 5.0 min. At 6.25 min, a transition to a predominantly fibrinolytic phase occurred. Equally to the product of the photothermal response (thermal coagula), thrombi that are resilient to breakdown contribute to vascular occlusion and thus likely to clinical results.

**Figure 9:**
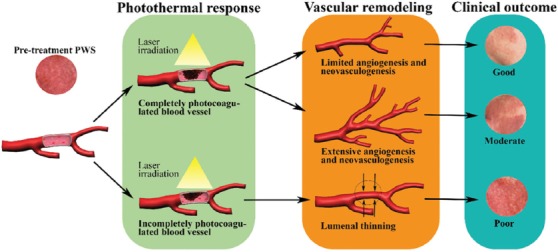
An overview of the differential photothermal responses to port wine stain (PWS) laser therapy and their respective vascular remodeling pathways in relation to clinical outcomes. Complete photo-occlusion (top pathway) most often results in vascular remodeling characterized by removal of the thermally afflicted vasculature followed by limited angiogenesis and/or neovasculogenesis. Inasmuch as the total dermal blood content is significantly reduced, these processes typically result in good clinical clearance after the vascular remodeling phase (“clinical outcome” panel, illustrating the changes in skin color before (left) and after (right) treatment). In the case of extensive angiogenesis or neovasculogenesis following laser treatment (middle pathway), the reduction in dermal blood volume is limited, corresponding to a moderate clinical result. Alternatively, particularly in refractory PWSs, light penetration is insufficient to induce complete photocoagulation of the vascular lumen, resulting in partial occlusion of the target vessels by a thermal coagulum (bottom pathway). During the remodeling phase, the thermal coagulum is either removed by recruited immune cells or becomes part of the vascular wall, leading to luminal thinning (small opposing arrows). This damage profile is associated with minimal reduction in dermal blood volume and hence poor clinical outcome.

Thrombus formation is followed by a remodeling phase (thrombus organization). During this process, vascular repair or reperfusion can occur, which impedes therapeutic efficacy and results in post-treatment lesional recurrence. Two possible vascular remodeling processes capable of restoring perfusion after PDL irradiation have been suggested: angiogenesis, promoted by a drop in tissue oxygen tension, and neovasculogenesis, which embodies recanalization of the thrombus by proteolytic and phagocytic activity from monocytes and macrophages and transdifferentiation of monocytes and endothelial progenitor cells to mature ECs (Figure 9) [144,158]. Note that reperfusion as a result of the same or similar processes also hampers clinical results in completely photocoagulated vessels [159,160]. Clinical PWS studies with angiogenesis inhibitors (or other pharmacological adjuvants) are summarized in Supplemental Table S3.

## 3. Site-specific pharmaco-laser therapy

**Figure 10:**
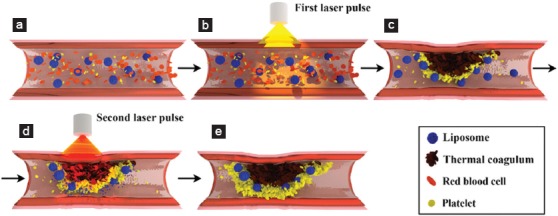
The principles of site-specific pharmaco-laser therapy are shown in an incompletely occluded laser-treated port wine stain (PWS) vessel. During thrombus development (c), procoagulant and/or antifibrinolytic liposomes accumulate in the thrombus. After induction of drug release, for example, by mild hyperthermia generated by a second laser pulse (d), the thrombomodulators and antifibrinolytic agents are activated/released. This promotes thrombus development and deters thrombus breakdown (e) and is expected to lead to complete vascular occlusion and enhanced therapeutic efficacy.

As shown in Figure 3, the efficacy of the gold standard treatment is relatively poor and warrants the development of novel therapeutic strategies beyond the available laser setting permutations and ancillary technologies, which include perioperative epidermal cooling. Accordingly, a novel treatment modality is being developed on the basis of what is known about laser-tissue interactions and the photophysical and biochemical responses that underlie the suboptimal response to SP. Figures 2 and 5 illustrate the two primary processes that dictate vaso-occlusion in incompletely-photocoagulated vasculature, namely the photothermal and the hemodynamic response. As stated, the photothermal response is a static process that cannot be modulated, whereas the hemodynamic response is dynamic and therefore amenable to pharmacological intervention. Based on the premise of exacerbating the hemodynamic response to achieve full occlusion in PWS vessels (i.e., the clinical aim that corresponds to a good clinical outcome), SSPLT combines laser therapy with the use of pharmaceutical agents that promote thrombosis or reduce fibrinolysis (Figures 5 and 10).

### 3.1. Drug delivery systems for SSPLT

To pharmacologically promote thrombosis, procoagulant and antifibrinolytic drugs can be employed. It is, however, imperative that these compounds do not result in systemic alterations in the hemostatic ’checks and balance’ system inasmuch as perturbations in this finely-tuned system could lead to hazardous complications, such as deep venous thrombosis, infarction, or hemorrhage. To prevent the occurrence of such adverse events, SSPLT employs a drug delivery system (DDS) with stable physicochemical properties and minimal passive release of the encapsulated drug over time, targeting capacity to the site of laser-induced damage, an efficacious drug release mechanism, and low immunogenicity [161,162].

#### 3.1.1. Liposomal drug delivery systems

**Figure 11:**
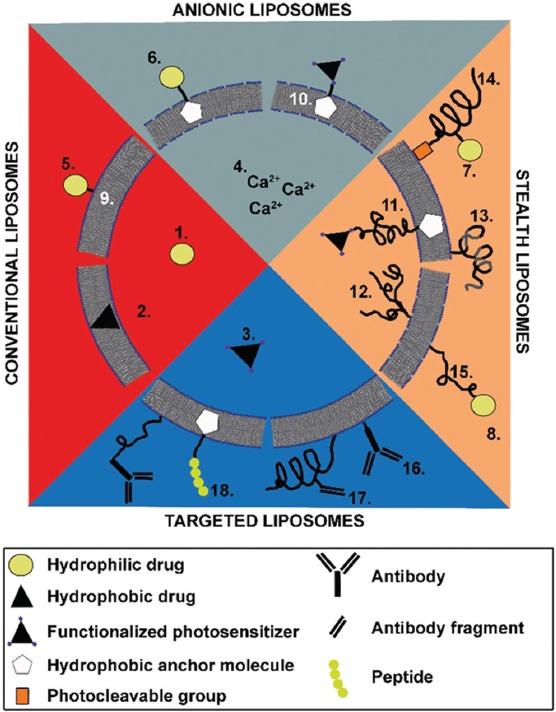
Schematic overview of possible liposomal formulations for site-specific pharmaco-laser therapy. These can be divided into four (potentially overlapping) categories: conventional liposomes, anionic liposomes, sterically stabilized liposomes, and targeted liposomes. Each main category may encompass any of the following subcategories: (I) types of drugs: 1 - hydrophilic drugs (e.g., tranexamic acid); 2 - hydrophobic drugs (e.g., photosensitizers); 3 - functionalized hydrophobic drugs (e.g., functionalized photosensitizers); 4 - ions (e.g., calcium); (II) drug-grafting methods: 5 - (covalent) attachment to a component (phospho)lipid; 6 - (covalent) attachment to an anchor molecule (e.g., cholesterol); 7 - (covalent) attachment to a polymer side chain (e.g., PEG); 8 - (covalent) attachment to a functionalized distal end of a polymer; (III) membrane composition: 9 - phosphatidylcholines; 10 - phosphatidylcholines with a molar fraction of anionic/cationic (phospho)lipids; (IV) methods of steric stabilization: 11 - single chain polymer (e.g., PEG); 12 - multichain polymer; 13 - multiblock copolymer (e.g., di- or triblock copolymers); 14 - photocleavable polymers (e.g., PEGylated plasmalogens); 15 - adsorbable polymers (onto anionic/cationic membrane surface); (V) methods of targeting: 16 - antibodies; 17 - antibody fragments (e.g., antigen-binding fragments or nanobodies); and 18 - peptides. The main categories are not mutually exclusive; e.g., sterically stabilized liposomes may contain anionic membrane constituents as well as antibodies for targeting. Figure adapted with permission from Aguilar et al. [103].

Liposomes, polymeric drug carriers, cells, and cell ghosts are all potentially suitable DDSs for SSPLT. Liposomes, which are composed of phospholipids (Supplemental Table S4), encompass facile and scalable preparation techniques, manipulatable attributes (including heat-mediated drug release), low toxicity, and the ability to encapsulate hydrophobic and lipophilic molecules at high efficiency [163] (Figure 8). Phospholipids particularly suited for SSPLT are listed in Supplemental Table S5. Potential liposomal formulations are illustrated in Figure 11.

Rapid uptake of liposomes by the mononuclear phagocyte system (MPS) is prevented by adequately sizing the liposomes (0.16–0.21 μm) [164,165], thereby increasing in vivo circulation times. Particle surface charge (zeta-potential) also governs the liposome elimination rate: high (positive or negative) surface charge corresponds to shorter circulation times and uptake by various cell types [166]. Liposome bilayer properties such as fluidity (i.e., cholesterol content) can also influence particle uptake. Readers are referred to other reviews [167-169] for more in-depth information regarding the effects of liposome physicochemical properties on particle-cell interactions.

In addition, liposomes (or other drug carriers) can be sterically stabilized, which is typically performed by grafting of polyethylene glycol (PEG) onto the carrier’s surface [170,171]. For liposomes, the PEG polymers are usually linked to phosphatidylethanolamine (PE) head groups of phospholipids. Although steric stabilization somewhat reduces the encapsulation efficiency of the DDS, it prevents liposome aggregation, which would impede proper sizing, and imposes so-called “stealth” properties that further reduce clearance by the MPS [172]. In case of PEGylation, stealth properties result from the repulsive effects of PEG polymers toward cell membranes, their hydrophilicity, and the decreased rate of (opsonizing) plasma protein adsorption [173,174]. The extent of PEG-mediated stealth effects is dependent on the size of the PEG polymers [175,176]. Steric stabilization can also be achieved by inclusion of covalently linked polymers, di- and/or multiblock copolymers, hydrophobized polysaccharides, polysialic acids, glucuronic acids, and/or (sialic derivatives of) gangliosides (Supplemental Table S6).

### 3.2. Triggered liposomal drug release mechanisms

To achieve rapid and localized drug release, a drug release mechanism needs to be incorporated into the DDS. A commonly used method is to impart thermosensitive properties on the liposomes. By carefully selecting the membrane lipid composition, thermosensitive liposomes can be made in which mild hyperthermia (~41–43 °C) induces an alteration in membrane permeability and consequently induces release of the cargo (as illustrated in Figure 6). Dipalmitoylphosphatidylcholine (DPPC) in particular is a popular phospholipid to confer thermosensitivity, as corroborated by its widespread use in thermosensitive liposome-based cancer treatments [177-179]. Modest improvements in release kinetics have been demonstrated by incorporation of lysolecithins [161,179]. Triggering of the DDS can be achieved by application of exogenous heat, i.e., using a heating pad or infrared light, or endogenous heat generated by a second laser pulse or light-emitting diode light at a wavelength attuned to the absorption maximum of the target chromophore, such as hemoglobin, water, or an administered ([co-]encapsulated) molecular absorber (e.g., indocyanine green [180], gold nanoparticles [181]).

Spatially and temporally controlled drug release could be facilitated by photo-oxidative modification of the liposomal lipid bilayer. It has been shown that laser irradiation of plasmenylcholine liposomes loaded with a photosensitizer induces membrane permeability [182]. Plasmalogens are glycerophospholipids characterized by the presence of a vinyl ether substituent at the *sn*-1 position of the glycerol backbone. The co-encapsulated photosensitizer produces ROS that cleave plasmalogens into single-chain surfactants, which subsequently accumulate and induce membrane defects. The photosensitizers zinc phthalocyanine, tin octabutoxyphthalocyanine, and bacteriochlorophyll a have previously been used for this purpose and were irradiated at 630–820 nm. The combination of PEG-modified plasmalogens and a photosensitizer can also be exploited to create photocleavable PEG polymers. This could constitute a useful method for a photoactivatable DDS in an alternative liposomal configuration, in which the pharmaceutical agents are incorporated into or bound to the liposomal surface, as PEGylation normally impedes accessibility of drugs to their target.

PE is a phospholipid that does not form stable liposomes at physiological pH and temperature [183]. Dioleoylphosphatidylethanolamine (DOPE) and 1,2-bis[10-(2`-hexadienoyloxy)decanoyl-*sn*-glycero-3-phosphocholine (bis-SorbPC)-containing PEG-liposomes (in a molar ratio of 3:1) can be photopolymerized to induce liposome fusion and trigger drug release [184]. Irradiation with ultraviolet (UV) light prompts cross-linking and separation of bis-SorbPC from other lipids. The obtained (DO)PE-enriched areas yield unstable liposomes that fuse, triggering drug release. Note that the use of UV light is undesirable for *in vivo* application considering its propensity to induce DNA mutations (phototoxicity) and poor tissue penetration. Alternatively, second or third harmonic light sources can be employed to resolve poor tissue penetration at short wavelengths, or more suitable molecular alternatives can be used. For example, green light (495–570 nm) can be used when 1,1′-dioctadecyl-3,3,3′,3′-tetramethylindocarbocyanine perchlorate is co-encapsulated [185]. A similar approach also exploits the destabilizing effect of PEs. Unlike PE, N-acylated PEs can form stable liposomes. Zhang and Smith have developed a PE-containing phospholipid with a photocleavable acyl-group: 6-nitroveratryloxycarbonylated 1,2-dioleoyl-*sn*-glycero-3-phosphoethanolamine (NVOC-DOPE) [186]. During UV light irradiation, the acyl group is removed, triggering liposome aggregation and fusion and thus drug release. Alternatively, Chandra et al. have used *o*-nitrobenzyl as a photolabile group to create UV light-photocleavable lipids [187].

Several photoactivatable DDSs have been developed based on photoisomerizable moieties, usually azobenzene. With azobenzene, UV light (~366 nm) promotes a trans-cis transformation leading to increased polarity and hydrophilicity, followed by membrane destabilization and drug release. Azobenzenes are particularly attractive because they feature reversible isomerization using visible light (> 420 nm). Several azobenzene-based lipid derivatives have been developed for the use in photosensitive liposomal DDSs [188,189]. Other previously employed photoisomerizable groups for liposomal release include spiropyran [190] and stilbene [191].

Photosensitizer-derived ROS can also directly induce liposomal drug release by photo-oxidation of unsaturated lipids, resulting in destabilization of the lipid bilayer and corollary drug release [181]. Photosensitizers produce ROS upon illumination at specific wavelengths. Pashkovskaya et al. have used trisulfonated aluminum phthalocyanine, glycerol-substituted zinc phthalocyanine, and chlorin e6 irradiated with red light to induce liposomal membrane permeabilization and cargo release [192]. Rwei et al. have already employed liposomes loaded with a near-infrared (NIR)-sensitive photosensitizer, 1,4,8,11,15,18,22,25-octabutoxyphthalocyaninato-palladium(II) (PdPC(OBu)_8_), in an *in vivo* rat model to facilitate on-demand sciatic nerve blockade [193]. The use of photosensitizers as procoagulants is further discussed in section 3.4.1.3.

As can be seen from the abovementioned options, many photoactivatable release systems rely on UV light irradiation. A relatively new method to reduce the adverse effects associated with UV light is the use of upconversion nanoparticles (UCNPs). These are capable of sequentially absorbing multiple photons of NIR light, which has relatively deep tissue penetration (in the order of millimeters to centimeters), and converting the photons into higher-energy light, such as UV, depending on their composition. Accordingly, Yao et al. have combined Tm3^+^ and Yb3^+^-doped NaYF4 UCNPs with 1,2-distearoyl-*sn*-glycero-3-phosphocholine liposomes incorporating azobenzene to form a DDS activated by NIR [194]. Additional investigations have to be performed to demonstrate the safety and efficacy of UCNPs *in vivo*.

### 3.3. Liposomal targeting strategies

Inasmuch as high drug concentrations are desired at the site of laser-mediated endovascular damage, but clinical doses of the administered prothrombotics and antifibrinolytics are preferably minimized, the DDS should be equipped with targeting capabilities to the sites of laser-induced thrombosis. Targeting can be achieved by the conjugation or grafting of antibodies, antigen-binding fragments, nanobodies, or immunoactive peptides to the surface of the DDS, as exemplified in Figure 11. An interesting example of the latter is the fluorophore-conjugated fibrin-binding peptide used by Weiss et al., which could enable liposome targeting and concurrently facilitate real-time visualization of liposome accumulation in the fibrin clot [195]. For PEGylated liposomes, antibodies can be attached to chemically-modified distal ends of the PEG chains [196].

With respect to targeting moieties, essentially any laser-induced and specifically expressed (plasma or membrane surface) molecule is suitable for liposome targeting. Accordingly, potential targets include molecules or epitopes expressed after activation of platelets, ECs, or the coagulation cascade, including GpIIb/IIIa (CD41) [197] and P-selectin (CD62P) [150,197] on activated platelets, E-selectin, intercellular adhesion molecule 1, and vascular cell adhesion protein 1 on ECs, and fibrinogen (RGD [198,199] and AGDV antibodies [200,201]) or, preferably, fibrin (fibrin-binding peptide [195]) in the developing fibrin clot. Naturally, it is imperative that binding of these molecules does not impair the extent of laser-induced thrombosis. Considering this caveat, our group demonstrated that antibody-based inhibition of CD62P using the rat anti-mouse RB40.34 clone did not affect the extent of laser-induced thrombosis in a hamster dorsal skin model [150].

Instead of laser-induced targets, the DDS could also exploit epitopes specifically expressed by or enriched in PWS vasculature, such as CD133 and CD166 [14].

### 3.4. Thrombosis- and fibrinolysis-modulating drugs

The liposome-encapsulated drugs may promote thrombosis by exerting an effect on platelet function (primary hemostasis), components of the contact activation and TF pathway (secondary hemostasis), or the fibrinolytic pathway (tertiary hemostasis). Furthermore, the drugs are required to be heat-stable and, provided that they are encapsulated in the aqueous compartment, small enough to permeate the membrane. Numerous drug candidates exist that meet these requirements and that can be employed for SSPLT.

#### 3.4.1. Primary hemostasis

The exacerbation of thrombosis can be pharmacologically modulated at all levels of hemostasis, as exemplified below.

3.4.1.1. Platelet agonists

The DDS could encapsulate compounds that target primary hemostasis, i.e., compounds that mediate platelet adhesion, activation, and/or aggregation. As described in section 2.2, laser-induced primary hemostasis is presumably triggered primarily by denatured proteins and exposure of the subendothelial matrix that ensues after endothelial damage and denudation. In situations with high shear rates, circulating or secreted von Willebrand factor (vWF) binds to the exposed collagen as well as the platelet GpIbα receptor to facilitate platelet rolling. Similar to a variety of other substrates, such as thrombin (generated during secondary hemostasis) and thromboxane A_2_ (TXA_2_), matrix components such as collagen, thrombospondin, and laminin act as potent platelet activators by binding to their cognate platelet receptors (e.g., glycoprotein V and VI), which activates platelets and triggers activation or expression of a variety of additional platelet receptors, platelet shape changes, selective release of α-granules, dense granules, and lysosomes (which can harbor prothrombotic factors such as vWF, platelet factor 4, thrombospondin, fibronectin, ADP, serotonin, polyphosphates, and calcium), and the synthesis and release of platelet activators (e.g., thrombin, TXA_2_ and platelet-activating factor [PAF]). Finally, platelet-platelet binding (i.e., platelet aggregation) is mediated by fibrinogen bound to activated GpIIb/IIIa molecules on adjacent platelets [202].

The DDS could incorporate natural or synthetic PAF phospholipids in the lipid bilayer or encapsulate ADP, serotonin, TXA_2_, or thrombin.

3.4.1.2. Calcium-containing liposomes

Extracellular calcium ions play an important role in the coagulation cascade, as they are essential for the formation of the FVIIa-TF-, tenase-, and prothrombinase complexes. Hu et al. [203] have shown that supraphysiological concentrations of extracellular calcium amplify ADP-induced platelet aggregation via a positive feedback mechanism involving TXA_2_ synthesis. Therefore, calcium can be suitable for (co-)encapsulation into the DDS that, upon triggered release (section 3.2.), would enhance coagulation and platelet aggregation.

3.4.1.3. Photosensitizers

As an alternative to conventional drugs that target primary or secondary hemostasis, which generally are expensive and heat labile, the DDS could also incorporate a photosensitizer in the lipid bilayer or the aqueous phase. Photosensitizers are currently being used for PDT of PWS (section 1.4). The thrombogenic effects that are beneficial for SSPLT result from ROS-induced endothelial damage, vasoconstriction, thrombus formation, and hemostasis [128,142,204].

Photosensitizers suitable for SSPLT include phthalocyanines, naphthalocyanines, and porphyrins from the group of chlorins and bacteriochlorins. The photosensitizers may be encapsulated in a separate liposomal formulation to comprise a DDS with procoagulant properties. As addressed in section 3.1, a photosensitizer could also be used to facilitate liposomal drug release.

#### 3.4.2. Secondary hemostasis

3.4.2.1. Tissue factor and the contact activation pathway

**Figure 12:**
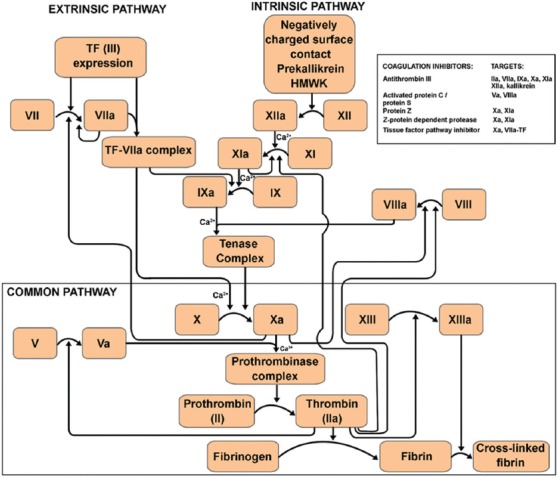
Overview of the coagulation cascade and a list of the endogenous factors that can modify coagulation at the indicated targets. In theory, all components represent possible targets that could be exploited in site-specific pharmaco-laser therapy to enhance the hemodynamic response following laser irradiation. Ca^2+^: calcium ions, HMWK: high-molecular-weight kininogen, TF: tissue factor.

The coagulation cascade embodies the TF and contact activation pathways; two series of zymogenic activations of circulating clotting factors that share a common final pathway in which prothrombin and fibrinogen are converted to thrombin and fibrin, respectively (Figure 12). Thrombin is also a strong platelet activator. The generation of fibrin is imperative for the stabilization of the primary platelet clot. Components of the coagulation cascade, such as FII(a), FIII, FV(a), FVII(a)-FXIII(a), (pre-)kallikrein, and high-molecular-weight kininogen could be included in purified or recombinant form in the DDS. Table 1 provides an overview of clinically available compounds that could be suitable for SSPLT.

**Table 1:** An overview of clinically available prothrombotic compounds.

Prothrombotic compound	Non-activated four-factor prothrombin complex concentrate	Activated prothrombin complex concentrate	Recombinant human factor VIIa	Fibrin glue
**Trade name(s), supplier(s)**	Kanokad, LFB; Octaplex^#^, Octapharma; Confidex/Beriplex^#^, CSL Behring; Cofact^#^, Sanquin	FEIBA, Baxter	Novoseven, Novo Nordisk	Tisseel/tissucol, Baxter; Beriplast, CSL Behring

**Component(s)**	Factor II, VII, IX, X + protein C (and in most forms^#^ protein S)	Mainly non-activated factor II, IX and X, mainly activated FVII, FVIII C:Ag	rFVIIa	Fibrinogen (factor I) and factor X + factor XIII, aprotinin, and calcium chloride (in Beriplast)

3.4.2.2. Coagulation inhibitor antagonists

A number of endogenous inhibitors restrain the coagulation cascade to proportionate the extent of coagulation. Therefore, antagonists of these endogenous inhibitors could also be included in the DDS to promote coagulation. The most prominent inherent inhibitors and their interaction with the coagulation pathway are addressed here.

Antithrombin III is a plasma-borne glycoprotein and serine protease inhibitor (serpin) synthesized by the liver that inactivates thrombin and inhibits FIXa-FXIIa and FVII as well as plasmin and kallikrein [205]. Its activity is markedly potentiated by heparin. Heparin cofactor II is another serpin that inhibits thrombin in the presence of certain glycosaminoglycans, such as heparin [206].

Protein C is an anticoagulant that is activated by thrombin-bound thrombomodulin on the EC outer membrane surface to form activated protein C (APC). In the presence of the cofactor protein S, APC inactivates FVa and FVIIIa [207]. Protein C is inhibited by protein C inhibitor whereas APC is inhibited by α1-protease inhibitor [206].

TFPI binds to FXa, thereby inhibiting it, and in this combination binds and inhibits the FVIIa-TF complex formed at the beginning of the TF pathway [208]. Protein S strongly potentiates this process as well [209].

Protein Z-dependent protease inhibitor, a blood-borne serpin, inhibits FXIa in the presence of calcium and, in conjunction with protein Z, FXa [210].

3.4.2.3. Anionic phospholipids

PS, an anionic phospholipid present in cell membranes [211], is an essential mediator of the conversion from prothrombin to thrombin [212,213]. In normal cells, including resting platelets, PS is asymmetrically distributed across the inner leaflet of the membrane [214-217]. An adenosine triphosphate-dependent amino-phospholipid-specific translocase maintains this membrane asymmetry [218,219]. Upon platelet activation (or apoptosis in any cell type), asymmetry is lost and PS is exposed on the platelet (or other cell types) outer membrane surface [218,220]. After activation, platelets also shed procoagulant microparticles that express PS on their surface [218]. Exposure of PS in the presence of calcium promotes coagulation via assembly of the prothrombinase complex, comprising FXa and FVa [153], which catalyzes the conversion of prothrombin to thrombin. Therefore, PS or other anionic phospholipids, such as phosphatidic acid and, to a lesser extent, phosphatidylglycerol and phosphatidylinositol [221], could be incorporated into the liposomal membrane to enhance coagulation.

3.4.2.4. Phosphatidylethanolamine liposomes

Klein et al. [222] have shown that very low-density lipoproteins (VLDL) can amplify the contact activation pathway by increasing FXII activity. The principal activating components in the VLDL membrane are PEs, a phospholipid [222] that contains a phosphoethanolamine head group (Supplemental Table S4). Hence, PE could be added to the DDS membrane to promote coagulation.

#### 3.4.3. Tertiary hemostasis

3.4.3.1. Antifibrinolytics

**Figure 13:**
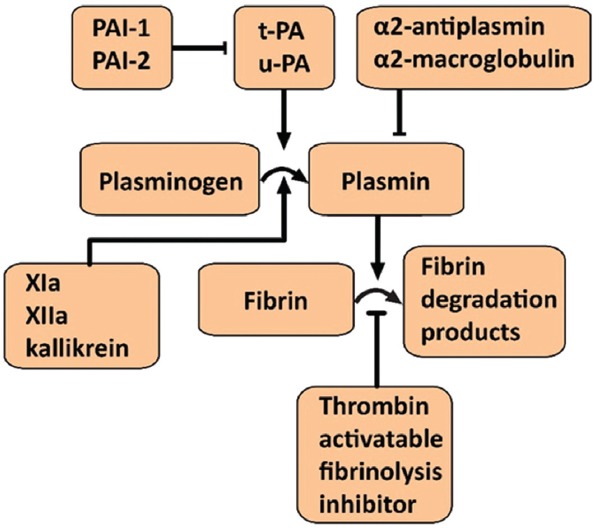
Overview of the fibrinolytic cascade and the endogenous factors that regulate this pathway. All components represent possible targets for inhibition of thrombus degradation that could be exploited in site-specific pharmaco-laser therapy. Abbreviations: PAI: plasminogen activator inhibitor, t-PA: tissue plasminogen activator, u-PA: urokinase-type plasminogen activator.

The fibrinolytic pathway, a cascade of serine proteases that mediate tertiary hemostasis, is activated in response to and simultaneously with the coagulation cascade and serves to counteract thrombus formation and restore luminal patency by the degradation of fibrin (Figure 13). During blood clot formation, zymogenic plasminogen incorporates into the clot by binding to exposed lysine residues in fibrin. Tissue plasminogen activator (t-PA) and urokinase-type plasminogen activator (u-PA), both synthesized by ECs, cleave plasminogen to its enzymatic active form plasmin. Subsequently, plasmin cleaves fibrin and fibrinogen into soluble degradation products, which results in lysis of the blood clot. Excessive activity of t-PA and u-PA is prevented by circulating plasminogen activator inhibitor 1 (PAI-1) and 2 (PAI-2), produced by hepatocytes and ECs [223].

The fibrinolytic system, considering its vital role in thrombus degradation, forms an important target for pharmacological modulation in SSPLT. Established drugs capable of inhibiting the fibrinolytic pathway are listed in Table 2. Tranexamic acid (TA) and ε-aminocaproic acid inhibit the biological activity of plasmin(ogen) by competitively binding the lysine binding sites of plasmin. Both are Food and Drug Administration-registered drugs and are very suitable for encapsulation into thermosensitive liposomes because of their small size, hydrophilicity, and high solubility at physiological pH (Table 2) [161,162]. Nevertheless, these drugs could also be co-infused in unencapsulated form since they exert an effect only in active thrombosis and pose a low risk for unwanted complications in their free form. The antifibrinolytic serpins aprotinin and nafamostat mesylate may be less suitable candidates inasmuch as these molecules also possess anticoagulant properties [224,225]. The DDS could also incorporate an inhibitor of uPA (receptors), a purified or recombinant agonist of PAI-1/2 or thrombin-activatable fibrinolysis inhibitor [226], a plasmin inhibitor, including α2-antiplasmin and α2-macroglobulin [223], and/or include plasmin-inhibiting long-chain fatty acids in the lipid bilayer, such as arachidonate, oleate, or stearate [227].

**Table 2:** Characteristics of the most common antifibrinolytics

Name	Tranexamic acid (TA)	ε-aminocaproic acid (ACA)	p-aminomethylbenzoic acid (AMBA)	4-aminomethyl-bicyclo-2,2,2-octane carboxylic acid (AMBOCA)	Aprotinin	Nafamostat mesylate
Trade name(s)	Cyclokapron, Lysteda	Hemocid, Amicar			Trasylol	Nafamostat, Futhan

Chemical structure	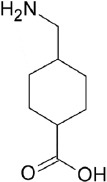	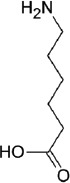	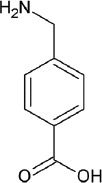	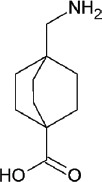	*	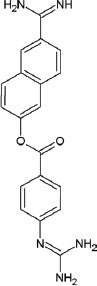

CAS ID	1197-18-8	60-32-2	56-91-7	24306-54-5	9087-70-1	81525-10-2

Molecular weight (g.mol^-1^)	157.2	131.2	151.2	183.2	6511.5	347.3

Water solubility (mg.mL^-1^)	167	505	9.890	#	> 10	0.0341

FDA UNII	37YD696II6	U6F3787206	68WG9JKC7L	#	04XPW8C0FL	1D2T74921W

* The chemical structure of aprotinin, a polypeptide with chemical formula C_284_H_432_N_84_O_79_S_7_, was not included because of its size. Readers are referred to https://pubchem.ncbi.nlm.nih.gov/compound/53487898 for structural details.

# Missing data. Abbreviations: *CAS ID*, Chemical Abstracts Service identifier; *FDA UNII*, Food and Drug Administration Unique Ingredient Identifier.

## 4. Discussion

Since the introduction of the current gold standard PDL therapy in the 1980s, much effort has been put into improvement of clinical outcomes by altering laser parameters (wavelength, pulse duration, and laser spot size), using different lasers, and the employment of new (ancillary) techniques such as multiple passes [228], epidermal cooling [102], and hypobaric pressure [229]. Despite technological advances (Figure 3), a considerable fraction of PWSs remains resistant to laser therapy [230] as the extent of photocoagulation is limited by a series of inevitable intrinsic factors described in section 1.3. The patients’ interest in improved therapies has been unwavering to date [21] and underscores the medical need for novel approaches to treat recalcitrant PWSs.

The current limitations of PWS treatment approaches reverberate the need for novel treatment modalities that employ alternative strategies to circumvent the inherent barriers discussed in section 1.3. SSPLT combines PDL with a procoagulant and antifibrinolytic DDS to promote the hemodynamic response, which is characterized by thrombus formation and contributes to vaso-occlusion in incompletely photocoagulated vessels, as a means to improve clearance rates in PWS patients. The prototype DDS employs two different liposomal formulations tailored to the procoagulant and antifibrinolytic components of the hemodynamic response. Both liposomal formulations consist primarily of DPPC and contain a molar fraction of DSPE-PEG2000 (PEG M_w_ = 2000 Da) [161,162] to prevent clearance by the MPS. The antifibrinolytic liposomes are loaded with TA and release the TA under mild hyperthermia in buffered solution [161] as well as whole blood [162] similar to heat-induced 5(6)-carboxyfluorescein release *in vivo* (Figure 6). The procoagulant liposomes contain a second-generation photosensitizer (metallated phthalocyanine) in the lipid bilayer [102] to induce thrombosis via locally produced ROS [128,231,232]. Preliminary proof-of-concept studies with similar formulations demonstrated that PEGylated DPPC liposomes encapsulating the photosensitizer zinc phthalocyanine or aluminum phthalocyanine in the phospholipid bilayer produce ROS upon irradiation with 671-nm laser light [233,234]. It was further shown that the photoproduced ROS are capable of oxidizing small molecules [233,235,236] and large proteins [236,237]. The liposomes were further capable of inducing cellular responses following illumination [125,235], which depended on the fluence rate [238], but were not affected by changing the membrane composition to impart a cationic charge on the liposome surface [124,237] or the conjugation of nanobodies to the distal end of PEG chains [196]. Both types of liposomes will need to be targeted to the site of laser-induced endovascular damage, which can be achieved by immunotargeting to platelet activation-dependent receptors such as CD62P (P-selectin) (section 3.3). Identically to PDT, liposomes require intravenous administration before laser irradiation.

Accordingly, the following clinical procedure is envisioned for SSPLT (Figure 10). The liposomal formulations are infused systemically in the PWS patient several minutes before lasing. Next, standard PDL treatment is performed to induce photocoagulation of PWS vessels and thrombosis in incompletely photocoagulated vasculature. After DDS accumulation in thrombi, the DDS can be triggered by a second laser pulse to induce (1) mild hyperthermia for the release of TA from thermosensitive liposomes and (2) a site-confined hyperthrombotic state from locally photoproduced ROS via thrombus-trapped, photosensitizer-containing liposomes. Both phenomena are expected to achieve complete and durable PWS vessel occlusion [145] and hence lesional blanching.

In the future, SSPLT could be combined with other novel techniques and therapies currently under investigation for combination with either PDL or PDT. Angiogenesis inhibitors, such as imiquimod and rapamycin, improve lesional blanching after laser therapy by preventing the replacement or repair of PWS vessels [159,239-243]. Another interesting development is the possibility to image and assess the microcirculation, and thus clinical efficacy, synchronously with laser therapy. These techniques currently include Doppler optical coherence tomography [244,245], photoacoustic imaging [246,247], laser speckle imaging [248-250], side stream dark field imaging [251], and orthogonal polarized spectral imaging [146].

Although our current focus is the use of SSPLT for PWSs, a similar treatment modality could be used to treat other vascular and vessel-related pathologies in the skin (hemangiomas, telangiectasias, pyogenic granulomas, venous lakes, and angiomas serpiginosum), eyes (choroidal neovascularization, retinal macro-aneurysms, intraocular melanomas, retinoblastomas, corneal vascularization, and central serous chorioretinopathy) and the gastrointestinal tract (e.g., blue rubber bleb nevus syndrome, gastric antral vascular ectasia, radiation proctocolitis, and hereditary hemorrhagic telangiectasia). Moreover, in oncology, this modality could be used for treatment of highly vascularized solid tumors and as a minimally invasive approach for complex arteriovenous malformations.

## 5. Conclusions

SSPLT is a novel development-stage treatment modality for PWSs that intends to address the limitations of PDL treatment in refractory PWSs by instilling complete vascular occlusion via a pharmacologically modulated hemodynamic response. This new strategy combines conventional PDL therapy with the prior administration of a liposomal DDS that contains prothrombotic and/or antifibrinolytic compounds that are locally activated and released, respectively. Clinical translation of SSPLT is expected to improve lesional blanching by inducing complete vascular occlusion in the PWS microcirculation that was insufficiently photocoagulated by the initial PDL treatment.
